# Assessment of the significance of patent-derived information for the early identification of compound–target interaction hypotheses

**DOI:** 10.1186/s13321-017-0214-2

**Published:** 2017-04-21

**Authors:** Stefan Senger

**Affiliations:** GlaxoSmithKline, Stevenage, Hertfordshire SG1 2NY UK

**Keywords:** Patents, Patent chemistry databases, SureChEMBL, ChEMBL

## Abstract

**Background:**

Patents are an important source of information for effective decision making in drug discovery. Encouragingly, freely accessible patent-chemistry databases are now in the public domain. However, at present there is still a wide gap between relatively low coverage-high quality manually-curated data sources and high coverage data sources that use text mining and automated extraction of chemical structures. To secure much needed funding for further research and an improved infrastructure, hard evidence is required to demonstrate the significance of patent-derived information in drug discovery. Surprisingly little such evidence has been reported so far. To address this, the present study attempts to quantify the relevance of patents for formulating and substantiating hypotheses for compound–target interactions.

**Results:**

A manually-curated set of 130 compound–target interaction pairs annotated with what are considered to be the earliest patent and publication has been produced. The analysis of this set revealed that in stark contrast to what has been reported for novel chemical structures, only about 10% of the compound–target interaction pairs could be found in publications in the scientific literature within one year of being reported in patents. The average delay across all interaction pairs is close to 4 years. In an attempt to benchmark current capabilities, it was also examined how much of the benefit of using patent-derived information can be retained when a bioannotated version of SureChEMBL is used as secondary source for the patent literature. Encouragingly, this approach found the patents in the annotated set for 72% of the compound–target interaction pairs. Similarly, the effect of using the bioactivity database ChEMBL as secondary source for the scientific literature was studied. Here, the publications from the annotated set were only found for 46% of the compound–target interaction pairs.

**Conclusion:**

Patent-derived information is a significant enabler for formulating compound–target interaction hypotheses even in cases where the respective interaction is later reported in the scientific literature. The findings of this study clearly highlight the significance of future investments in the development and provision of databases and tools that will allow scientists to search patent information in a comprehensive, reliable, and efficient manner.

**Electronic supplementary material:**

The online version of this article (doi:10.1186/s13321-017-0214-2) contains supplementary material, which is available to authorized users.

## Background

A comprehensive and detailed knowledge of the biological targets a compound of interest interacts with is a key requirement for effective decision making in drug discovery. For formulating and substantiating hypotheses about such compound–target interactions, scientists gather proprietary information as well as information from the public domain. For the latter, publications in scientific journals and patents are the two main primary sources. As far as information derived from publications in scientific journals is concerned, there are now a number of well established databases in the public domain (e.g. ChEMBL [1]) that have widespread use when looking at compound–target interactions. However, the situation is very different for patents. Although there are now freely accessible patent-chemistry databases (e.g. SureChEMBL [2]), they are very much routed in the chemistry domain and do not tend to contain comprehensive bioannotations.^1^ In the case of SureChEMBL, a workflow for adding bioannotations (for biological targets and diseases) using the entity extraction engine TERMite [3] from SciBite has only recently been developed as part of the IMI-funded [4] Open PHACTS project [5] that ended in February 2016. This project has also made SureChEMBL accessible via the Open PHACTS API [6] which provides more programmatic access to patent-derived data and allows for relevant use cases to be addressed that cannot be efficiently tackled with a web-based GUI (e.g. SciFinder [7]). Notwithstanding the tremendous progress that has been made to make more patent-derived data easily and freely accessible, there is undoubtedly still a long way to go. At present there is still a wide gap between relatively low coverage-high quality manually-curated data sources (e.g. BindingDB [8], DrugBank [9]) and high coverage data sources that use text mining and automated extraction of chemical structures. For example, a recent study has come to the conclusion that only around 60% of chemical structures in patents are currently extracted successfully by the automatically generated patent chemistry databases IBM SIIP and SureChEMBL [10]. Similarly, more research is needed so that text mining for biological entities in patents reaches the same level of maturity that has already been achieved for text mining of the scientific literature [11]. The same is true when trying to address the hard problem of automated extraction of (meaningful) bioactivity data from patents. To be able to make a real difference in this area, further research and an efficient and sustainable infrastructure for making patent-derived data freely accessible to the scientific community is needed. However, funding to successfully achieve this will only become available if there is hard evidence for the significance of patent-derived information for future drug discovery efforts. Surprisingly little of such evidence has been reported so far. When studying the complementarity between public and commercial databases of bioactive compounds Southan et al. [12] found that in the largest commercial database that was included in their study only around 6% of compounds from patents overlapped with those appearing in scientific journals. Lowe and Sayle [13] report that compounds appearing in a set of patents for the period 2006–2013 and that are also part of ChEMBL19 can be found on average 345 days earlier in patents than in the scientific publication referenced in ChEMBL. To gather further such evidence, this study aims to quantify the relevance of patents for formulating or substantiating hypotheses for compound–target interactions. The specific question addressed here is how much earlier hypotheses for compound–target interactions can be formulated based on information from patents compared to using evidence derived from publications in scientific journals. Additionally, this study looks at how the answer to the question that is being addressed here changes when the ChEMBL database is being used as a substitute for the primary literature and SureChEMBL is being used as secondary source for patents. SureChEMBL contains more than 16 million compounds extracted from over 13 million patent documents from the USPTO, EPO and WIPO (United States Patent and Trademark Office, European Patent Office and World Intellectual Property Organisation, respectively) along with English abstracts of Japanese patents [2]. Although automated searches can assist in the initial stages of a study like this, careful manual processing and scrutiny is absolutely essential for obtaining reliable results. As a consequence of the inherent labour intensity, the number of compound–target interaction pairs that can be included in any one given study is rather limited and a potential unintended bias is almost impossible to avoid. However, considering the cumulative nature of the results a comprehensive picture can be built up over time through the publication of an increasing number of individual studies.

## Methods

To address the question of how much earlier hypotheses for compound–target interactions can be formulated based on information from patents compared to using evidence derived from publications in scientific journals, a reference list of compound–target interactions was compiled. To start with, known compound–target interactions for approved drugs as well as clinical and pre-clinical drug candidates were identified using a variety of sources, e.g. ChEMBL [1], DrugBank [9], Wikipedia, as well as selected reviews from the primary literature (e.g. Notte’s compilations of compounds entering Phase III clinical trials [14–16]). Only drugs and drug candidates with a molecular weight between 200 and 600 g/mol (for the parent compound) were considered. For each compound–target interaction pair an exact chemistry search (for the parent compound) in the primary literature was performed using SciFinder [7] as well as ChEMBL [1]. Subsequently, references were looked at individually to identify the earliest publication (in English) describing the compound–target interaction together with the chemical structure of the compound. Similarly, a patent search for the first WIPO, USPTO, or EPO patent application for a given compound–target interaction was performed. Patents that are not in English were only considered if it was possible to infer the compound–target interaction from the English title and/or abstract. Just to note that the process of deciding whether or not a given publication or patent ‘describes’ a specific compound–target interaction inevitably requires an element of judgement. In order to be included in the reference list, the publication date for the earliest patent describing a compound–target interaction had to be from the period 1990 onwards. To allow a fair comparison with the two secondary sources ChEMBL and SureChEMBL, compound–target interactions were only included in the reference list if the following three conditions were satisfied: a) the compound has a SureChEMBL ID, b) the compound has a ChEMBL ID, and c) the target has a ChEMBL target ID. This approach resulted in an initial list of 170 compound–target interactions for 130 drugs and drug candidates. 102 of the 130 compounds only appear in one compound–target interaction pair, 18 compounds appear twice, 8 compounds appear three times, and there are 2 compounds that appear in four compound–target interaction pairs. On closer inspection of the 65 compound–target interaction pairs for compounds that feature more than once in the reference list it became apparent that pairs sharing the same compound also shared the same patent and publication. To avoid a situation where these particular patents and publications bias the analysis a subset of 130 compound–target interaction pairs was produced where a given compound only appears in one interaction pair. Since it is not always obvious which of the targets is most relevant this was simply done by arbitrarily choosing the target with the smallest Entrez Gene ID [17]. The resulting manually-curated list of 130 compound–target interactions together with the corresponding patents and publications was used as reference list in this study (see Additional files 1, 2). The patents in the reference list cover the period May 1990–September 2013 and the publications cover the period December 1992–October 2015. Just to note that there are many more compound–target interactions that satisfy the criteria described above. Due to the necessary manual processing involved in reliably establishing the first scientific publication and patent only a limited number of pairs could be chosen for this study. As an inevitable consequence of the limited size it has to be accepted that it is impossible to ensure that the final selection of compound–target pairs is free of any unintentional bias (e.g. in regards to target classes, companies). All details about the reference list are being shared (see Additional file 1) to allow full scrutiny.

Searches in ChEMBL and SureChEMBL were executed through the freely accessible API version 2.1 [6] of the Open PHACTS Discovery Platform [18–21]. Automation of the queries and processing of the results was performed with the help of the workflow tool Pipeline Pilot [22] from BIOVIA. First, the ‘Chemical Structure to URI’ API call was used to convert the parent SMILES string to the Open PHACTS compound URI (Uniform Resource Identifier). This compound URI was then passed as input to the ‘Patents for Compound: List’ API call to retrieve a list of patents associated with the compound of interest. Lastly, the ‘Patent Entities: List’ API was called to retrieve a list of all biological targets for a given patent. To identify the earliest publication for a compound–target interaction pair in ChEMBL, the ‘Compound Pharmacology: List’ API call was performed using the Open PHACTS compound URI as input. The response to this API call contains information about the biological target and the scientific publication which allows filtering for the publication of interest. For the cases where the patent or publication in the reference list was not found by the automated workflow, subsequent manual searches using SureChEMBL at http://www.surechembl.org and the ChEMBL GUI at http://www.ebi.ac.uk/chembl (ChEMBL22) were also performed.

Extensive manual searches were performed for all compound–target interaction pairs using a combination of SciFinder [7], PubMed [23] and the SureChEMBL and ChEMBL GUIs. All primary journal or patent references were looked at individually to confirm that they contain the structure of the compound and the target in question is mentioned. Just to note that as with all approaches using manual curation, an element of subjectivity is unavoidable. The results from the manual searches were used for the subsequent analysis.

## Results

The reference set compiled here covers situations where a patent and a publication describing a specific compound–target interaction were published almost simultaneously as well as examples where a patent was published many years before a publication. For instance, in the case of the Endothelin receptor antagonist Atrasentan [24] the patent WO-1996006095-A1 was published on 29 February 1996 and just one day later an article was published online in the Journal of Medicinal Chemistry. An example from the other end of the spectrum is the drug candidate Crenolanib that inhibits platelet-derived growth factor receptors (PDGFR) [25]. A patent linking the structure of Crenolanib to PDGFR was published as early as March 2004 whereas the chemical structure of Crenolanib could only be linked to PDGFR through a publication in April 2013, more than 9 years later. Crenolanib is an example of the situation where earlier publications mention a compound name and the target but the chemical structure is not given (e.g. the publication [26] with the Pubmed ID 19738123 in September 2009 mentions CP-868,596, a synonym for Crenolanib, but no structure was specified).

**Fig. 1:**
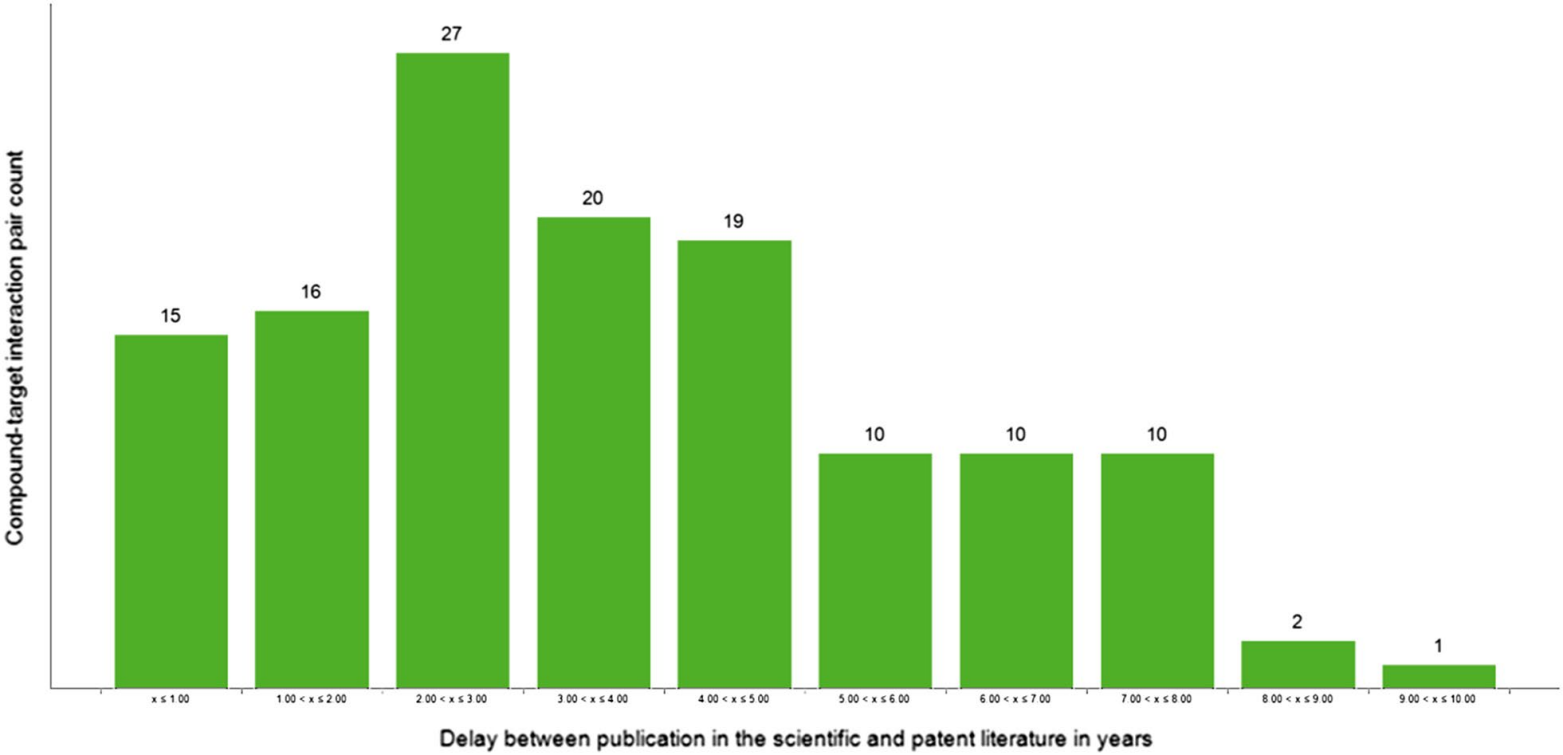
Binned delay between publication in the scientific literature after appearing in a patent in years. For each bin the number of compound–target interactions pairs is given

**Fig. 2:**
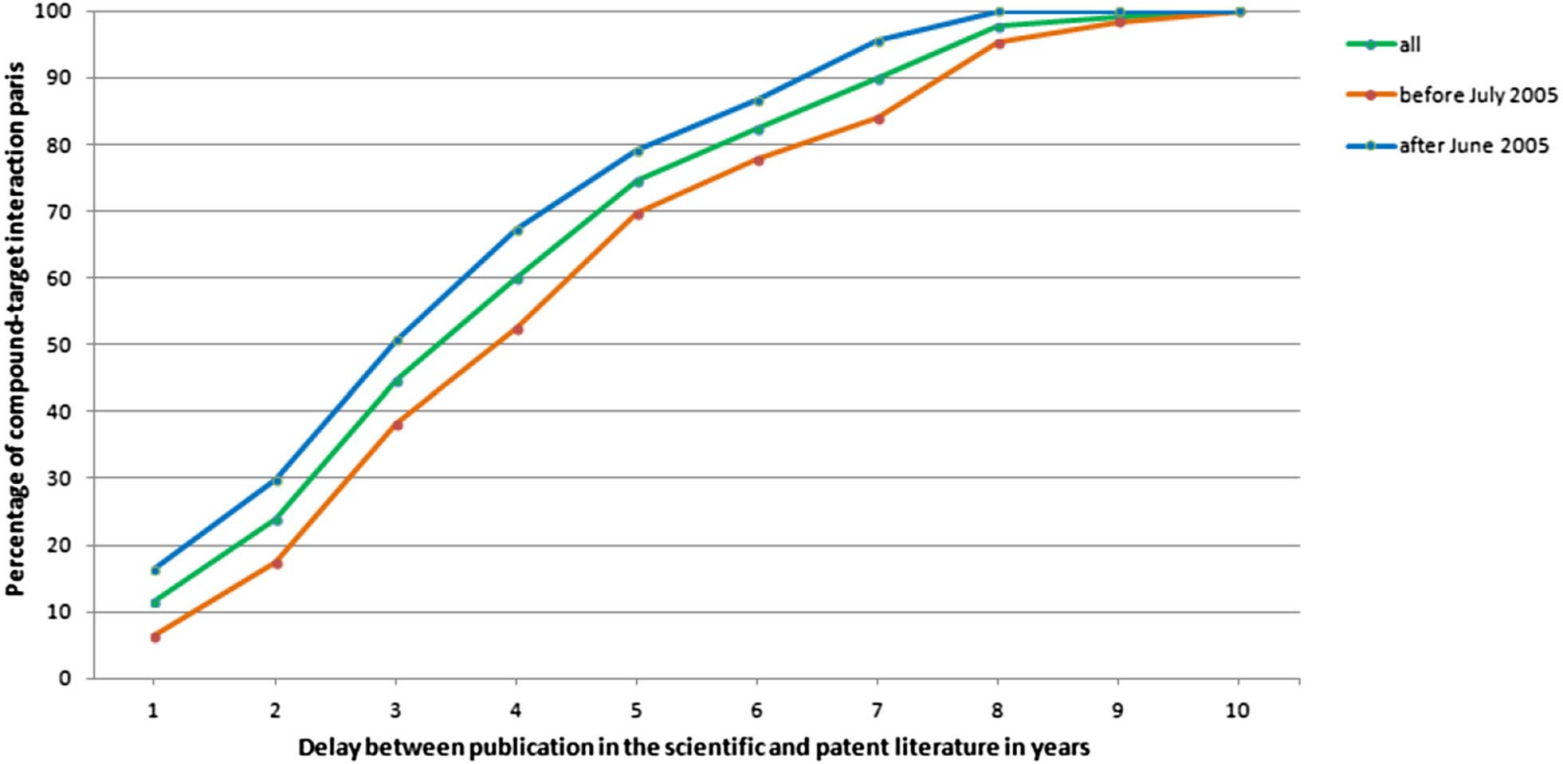
Delay between publication in the scientific literature after appearing in a patent. Percentage of compound–target interaction pairs from the reference set for which the delay between publication in the scientific literature after appearing in a patent is less or equal to a given number of years (*green* all 130 interaction pairs, *orange* 67 pairs with patents published before July 2005, *blue* 63 pairs with patents published after June 2005)

Figures 1 and 2 summarise the findings in regards to the question of how much earlier hypotheses for the 130 compound–target interactions in the reference set can be formulated based on information from patents compared to using evidence derived from publications in scientific journals. As can be seen in Fig. 1, for only 15 of the interaction pairs from the reference set a publication referring to the compound–target interaction appears in a scientific journal within one year after the publication of the patent. The delay between publication in a scientific journal after publication in a patent is 2 years or less for only around 24% (cf. Fig. 2, green) of the interaction pairs. In order to be able to identify 60 or 80% of the interaction pairs from the scientific literature one has to accept delays between publication in the scientific and patent literature of up to 4 or 6 years, respectively (cf. Fig. 2, green). The average delay across all 130 compound–target interaction pairs is 3.7 years.^2^

In order to study whether or not the delay might be dependent on how old a patent is the reference set was split in roughly half (i.e. 67 pairs with patents published before July 2005 and 63 pairs with patents published after June 2005). Figure 2 shows the results for the entire set of 130 pairs (green) as well as the results for pairs with patents before July 2005 (orange) and with patents after June 2005 (blue), respectively. As can be seen, it appears that the gap between publication in the scientific literature after patent publication is smaller for the compound–target interaction pairs from the set that contain patents that were published after June 2005. The observed difference is about 1 year, e.g. it takes 3 or 4 years after the initial patent publication date before approximately 50% of the interaction pairs can also be found in the scientific literature depending on whether the pairs contain patents published before July 2005 or after June 2005, respectively.

Next, the question of what impact it has when SureChEMBL is used as secondary source for patent information was investigated (see Additional file 3 for results). A comparison between the patents in the manually-curated reference list and the patents found in SureChEMBL showed that for 93 of the 130 compound–target interaction pairs (i.e. 71.5%) the earliest patents (as captured in the reference list) were found by the searches in SureChEMBL. From the remaining 37 interaction pairs it was only possible for 21 pairs to find patents that were published before the publication in the scientific literature (as captured in the reference list). For these 21 pairs the average time between the publication in the scientific literature after the publication of the patent was reduced from 5.3 years (based on the reference list) to 2.9 years (when using SureChEMBL). For the remaining 15 pairs the searches in SureChEMBL only resulted in patents that were published after a publication in the scientific literature appeared.

An analysis of the reasons why for 37 of the 130 interaction pairs the patents from the reference list were not found when using SureChEMBL as secondary source led to the finding that for 28 of the pairs the chemical structure for the compound in the interaction pair was not correctly represented in SureChEMBL and hence the chemistry search did not retrieve the patent from the reference list. This is broadly in line with what one would expect based on an earlier finding indicating that the SureChEMBL workflow successfully extracts only around 60% of chemical structures in patents [10]. For the remaining 9 interaction pairs bioannotation-related reasons led to the fact that relevant patents were missed. For example, in the patent WO-2001090076-A1 “PDE4” as well as “phosphodiesterase-4” is mentioned and the patent is annotated with the gene PDE4A. However, in the patent US-5712298-A PDE4 is only referred to as either “PDE IV” or “PDE-IV” and the bioannotation workflow developed by the Open PHACTS project does not seem to have annotated this patent with the gene PDE4A. For the majority of the other 8 interaction pairs the relevant gene was not successfully annotated by the implementation of the text mining workflow used by the Open PHACTS project since the patent documents do not refer to specific genes but only reference the biological targets in more generic ways (e.g. HDAC1 was only referred to as “histone deacetylase”).

Finally, it was investigated what impact it has when ChEMBL22 is used as secondary source for publications from the scientific literature (see Additional file 3 for results). For the 130 compound–target interaction pairs in the reference list the earliest publication in the scientific literature that was found by the manual search was also found in ChEMBL for 60 (i.e. 46.2%) of the pairs. For 67 of the remaining 70 interaction pairs the publication in the reference list is not part of the document corpus of ChEMBL. A detailed analysis showed that 31 of the 67 publications appear in journals that are not represented in ChEMBL at all whereas the remaining 36 appear in journals that have at least some representation in the ChEMBL document corpus even though it might only be very small. For example, there are only approx. 100 publications from the Journal of Pharmacology and Experimental Therapeutics in ChEMBL (compared to the approx. 20,000 publications each from the Journal of Medicinal Chemistry and Bioorganic Medicinal Chemistry Letters. These two journals together form approx. 60% of the journal corpus in ChEMBL). The publications for 3 of the interaction pairs were not found in ChEMBL because no bioactivity data was included for these compounds in the publications.

**Fig. 3:**
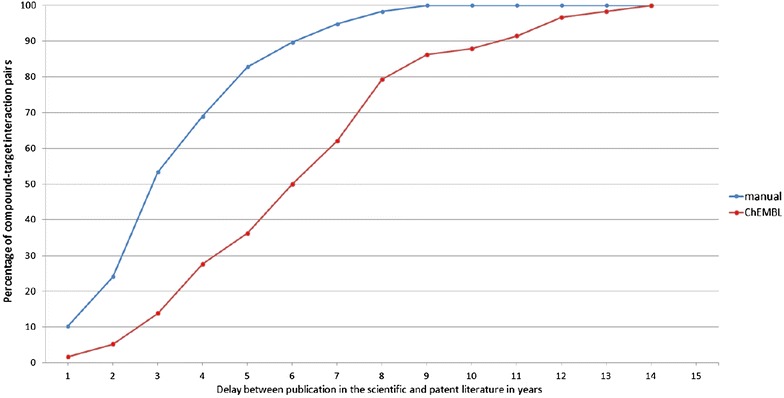
Effect of using ChEMBL as secondary source. Percentage of the 58 compound–target interaction pairs from the reference set (where the publication found in ChEMBL is not the earliest publication) for which the delay between publication in the scientific literature after appearing in a patent is less or equal to a given number of years (*blue* publication from manual search vs. patents, *red* publication from ChEMBL vs. patents)

For 58 of the 70 interaction pairs where the publication in the reference list is not part of the document corpus of ChEMBL alternative publications in ChEMBL could be found. The ‘penalty’ in terms of the additional time it takes before a compound–target interaction pair can be found in ChEMBL is shown in Fig. 3. Whilst one has to wait approx. 3 years after the publication of a patent before half of the 58 compound–target interaction pairs can also be found in the scientific literature, the gap increases to 6 years if ChEMBL is used as a secondary source for publications in the scientific literature. The situation is similar when 80% is used as a threshold since one has to wait for 8 years instead of approx. 5 years (i.e. an additional 3 years) if ChEMBL is used instead of performing a search in the primary literature.

## Discussion

The observation that only a small percentage of compounds reported in patents also appear in publications in scientific journals might already be seen as sufficient evidence to conclude that patents are a key source of information for drug discovery [12]. Furthermore, since novel compounds are usually disclosed in patents before they appear in the scientific literature it seems prudent to harvest information from patents even for cases where compounds are reported in both patents and scientific journals. Hence, one might argue that it is less a question of whether or not patents should always be included when scientists perform searches for information in the public domain but rather a question of how scientists can perform searches in the patent literature in an comprehensive, reliable, and efficient way. The latter challenge is far from being fully addressed. The sheer size and rate of growth of the patent corpus makes it unrealistic to even attempt a comprehensive coverage if manual extraction of information from patents is being used. Consequently, many manually-curated sources have a particular focus, e.g. on specific classes of biological targets, or might limit the number of chemical compounds per patent. Whilst patent database that are generated through automated extraction of entities from patents are well placed to achieve a good coverage, they are currently still in their infancy and reliability remains a challenge (see for example [10]). The lack of efficient ways for performing searches is particularly limiting in the public domain. For example, the EBI does so far not provide an API to query SureChEMBL. Through the creation of the Open PHACTS Discovery Platform, the Open PHACTS project created the first freely accessible API that allows to search SureChEMBL for chemical structures, biological targets and diseases. However, based on the complexity and scale of the task in hand it is clear that there is a long way to go and further significant investments are needed to reach a point where scientists can and will use patent-derived information in their day-to-day decision making. In order to create compelling incentives for funding bodies and companies to make the necessary investments in further research and development in this area there has to be a clear understanding of the resulting benefits for drug discovery. Surprisingly, not much has been reported so far that assists in quantifying the positive impact of greater access to patent-derived information on decision making in drug discovery. Furthermore, there has been a strong focus on chemistry, e.g. in the study of Lowe and Sayle [13] who looked at the question of how much earlier compounds appear in patents before they can also be found in scientific publications. Their results indicate that the time gap between patents and scientific journals might be something in the region of one year. The aim of this study was to build on the existing findings by quantifying the benefits of using patent-derived information when looking at compound–target hypotheses. After all, one might argue that creating such links between compounds and biological targets is the essence of drug discovery. To achieve the aim of this study, a manually-curated list of 130 compound–target interaction pairs was compiled. The analysis of this list revealed that in stark contrast to what has been reported for novel chemical structures, only about 10% of the compound–target interaction pairs reported in the patent literature were also found in publications in the scientific literature within one year. The average delay across all interaction pairs is close to 4 years. In the set used in this study the observed ‘time penalty’ is smaller for more recent patents compared to patents published before July 2005 but with an average of around 3.3 years it is still significant.

In an attempt to benchmark current capabilities, this study examined how much of the benefit described above can be retained when the bioannotated version of SureChEMBL (that is accessible through the Open PHACTS API) is used as a secondary source for the patent literature. Encouragingly, this approach found the patent in annotated set for 72% of the compound–target interaction pairs. Only for 12% of the pairs the search in SureChEMBL did not find a patent that was published before a publication in a scientific journal. For a database that is generated by automated entity recognition (of chemical structures and biological concepts) this is a very encouraging result. At this point it seems appropriate to note that although at the time of writing the bioannotated version of SureChEMBL used for this study is freely accessible through the Open PHACTS API (provided by the Open PHACTS Foundation [27]), future availability should not be taken for granted.

Although the primary focus of this study is information derived from patents, it seemed obvious to also investigate the effect of using the database ChEMBL as a secondary source for the scientific literature. Based on the fact alone that ChEMBL is a bioactivity database, it is to be expected that a search in ChEMBL for a given compound–target interaction pair will not necessarily find the earliest publication in the scientific literature. Indeed, what was observed was that the publication from the annotated set was only found for 46% of the 130 compound–target interaction pairs when ChEMBL was searched. The reason that the publications for over half of the interaction pairs were not found is almost exclusively due to the fact that the respective publications are not part of the ChEMBL22 document corpus. Hence, it appears that the ‘penalty’ of using ChEMBL as a secondary source is greater than what was observed for SureChEMBL. This finding can be viewed as a reminder that there rarely is a ‘one size fits all’ data source. Whilst ChEMBL is an excellent source for bioactivity data, it might not have the desired coverage when the objective is to generate compound–target interaction hypotheses and it is seen as acceptable to use evidence other than experimental bioactivity data.

## Conclusions

Patent-derived information is a significant enabler for formulating compound–target interaction hypotheses even in cases where the respective interaction is reported in the scientific literature. Results obtained for a manually-curated list of 130 compound–target interaction pairs indicate that on average interaction hypotheses can be formulated in the region of 3–4 years before publication in a scientific journal. These findings clearly highlight the significance of future investments in the development and provision of databases and tools that will allow scientists to search patent information in a comprehensive, reliable, and efficient manner. The more scientists have access to these databases and tools the greater the impact will be on the discovery of new effective medicines.

## Additional files

**Additional file 1.** Manually curated set of 130 compound–target interaction pairs annotated with the earliest patent and publication.

**Additional file 2.** InChI keys for all compounds in the manually curated set.

**Additional file 3.** Earliest patent in SureChEMBL and earliest publication in ChEMBL for the 130 compounds from the annotated set.

## Abbreviations

API: application programming interface
EPO: European Patent Office
GUI: graphical user interface
URI: Uniform Resource Identifier
USPTO: United States Patent and Trademark Office
WIPO: World Intellectual Property Organization

## Footnotes

1 BindingDB [8] contains some protein–ligand affinity data extracted from recent US patents. The target is to grow the patent-derived affinity data by approx. 35 k measurements per year.

2 To provide an indication of how much the average delay might depend on the size and composition of the set used here, all 130 interaction pairs were sorted by the delay in years in an ascending order and the average delay for the first 100 interaction pairs was calculated. The average delay of 2.8 years for this subset of 100 interaction pairs (with the smallest delays) is still significantly larger than what was, for example, reported by Lowe and Sayle [13].
